# The Impact of Physical Training on Circulating Retinol-Binding Protein 4: A Systematic Review

**DOI:** 10.33549/physiolres.935730

**Published:** 2026-04-01

**Authors:** Zaki ALSAHAFI, Ahmaed BAASHAR

**Affiliations:** 1 Department of Basic Sciences, College of Science and Health Professions, King Saud bin Abdulaziz University for Health Sciences, Jeddah, Saudi Arabia; 2 King Abdullah International Medical Research Center, Jeddah, Saudi Arabia

**Keywords:** Retinol-Binding Protein 4 (RBP4), Exercise training, Aerobic exercise, Resistance training, Obesity, Type 2 diabetes mellitus (T2DM), Insulin resistance, Adipokines, Metabolic health

## Abstract

Obesity and type 2 diabetes mellitus (T2DM) are strongly associated with insulin resistance and chronic inflammation. Retinol-Binding Protein 4 (RBP4), an adipokine secreted by the liver and adipose tissue, has been implicated in metabolic dysfunction, with elevated circulating levels linked to impaired glucose homeostasis. Exercise is known to improve insulin sensitivity; however, its effects on RBP4 remain unclear. This systematic review aimed to synthesize the available evidence on the impact of exercise interventions on circulating RBP4 concentrations and to evaluate differences by exercise modality and population characteristics. The review followed PRISMA guidelines. A systematic search of PubMed, Web of Science, and Google Scholar (2005–2025) was conducted. Inclusion criteria were human studies ≥18 years evaluating structured aerobic, resistance, or combined training with pre- and post-intervention RBP4 measurement. Non-exercise interventions and animal studies were excluded. Data extraction and quality appraisal were independently performed using Joanna Briggs Institute checklists. Out of 1,422 records screened, 16 studies met the eligibility criteria, including randomized controlled trials, quasi-experimental studies, and pre–post designs. Participants included healthy individuals, obese subjects, and patients with T2DM or metabolic syndrome. The exercise interventions varied from a single session to structured aerobic or combined aerobic-resistance programs lasting up to 12 weeks. Results showed context-dependent effects with conflicting results between different studies. Aerobic and combined aerobic-resistance exercise were associated with significant reductions in circulating RBP4 levels among obese and T2DM groups, whereas results in healthy individuals remained inconsistent. In contrast, single-session and endurance training interventions did not produce significant effects. Exercise training demonstrates potential to lower circulating RBP4, particularly in metabolically compromised populations. However, inconsistent results highlight the need for larger, standardized trials to clarify exercise modality-specific effects.

## Introduction

Obesity is a chronic, multifactorial disease marked by excessive fat accumulation, particularly in visceral tissue, and is closely linked to higher risks of cardiovascular disease and type 2 diabetes mellitus (T2DM) [1]. T2DM is defined by hyperglycemia due to insulin resistance and inadequate insulin secretion. Projections indicate that by 2050, over 1.3 billion individuals will be affected by diabetes, with age-standardized prevalence exceeding 10 % in most countries. Over 90 % of cases will be T2DM, with obesity as the leading risk factor [2]. Both obesity and T2DM contribute to chronic low-grade inflammation, driven by elevated levels of pro-inflammatory cytokines and immune cells [3,4]. Retinol-Binding Protein 4 (RBP4) is an adipokine secreted by adipocytes and hepatocytes, originally identified as the only specific transport protein for retinol (vitamin A) in the circulation [5,6]. In addition to its function in retinol transport, RBP4 has been implicated in the regulation of glucose metabolism and insulin sensitivity [7,8]. It has been shown that elevated RBP4 levels impair cellular glucose uptake and attenuate glucose signaling [9]. Earlier studies showed that circulating RBP4 levels are elevated in patients with T2DM [10–14]. In 2005, it was first reported that increased RBP4 is directly associated with insulin resistance and glucose intolerance [7]. This was later confirmed in human clinical studies which showed that elevated circulating RBP4 levels were strongly associated with insulin resistance and obesity [8,15,16]. Additionally, decreased RBP4 levels achieved through medication, lifestyle interventions or weight loss is associated with improved insulin sensitivity and better metabolic outcomes [17,18].

It is well established that exercise training can prevent T2DM by improving insulin sensitivity and maintaining glucose homeostasis [19,20]. Evidence from previous studies suggests that this effect may be partially mediated through reductions in circulating RBP4 levels [8,21,22–29]. This gives rise to an interesting hypothesis: exercise induces anatomical and functional adaptations in adipose tissue, liver, skeletal muscle, and the pancreas [30], which in turn could modulate RBP4 secretion. Altered RBP4 levels subsequently influence insulin sensitivity, thereby linking exercise-induced tissue adaptations to metabolic regulation. However, the influence of exercise on circulating RBP4 levels is inconsistent, with variability across study populations and training modalities. Such differences may reflect the specific effects of exercise types on skeletal muscle anatomy, including modifications in fiber type composition, vascularization, and mitochondrial density which in turn could affect RBP4 differently. To the best of our knowledge, no systematic review has previously assessed the impact of structured exercise training on circulating RBP4 levels. Therefore, this systematic review aimed to evaluate the impact of exercise training on circulating RBP4 levels and to examine how different exercise modalities influence RBP4 responses across populations. The findings will offer an up-to-date critical synthesis of the evidence, refine exercise recommendations for obesity and T2DM, and enhance understanding of the molecular pathways linking exercise, circulating RBP4, and metabolic health.

## Methods

This systematic review was conducted according to the Preferred Reporting Items for Systematic Reviews and Meta-Analyses (PRISMA) guidelines [31]. The study protocol is registered with the International Prospective Register of Systematic Reviews (PROSPERO) (ID: CRD420251052611). This study was approved by the King Abdullah Medical Research Center (KAIMRC) Institutional Review Board in Jeddah, Saudi Arabia (study number: NRJ25/011/6).

### Literature screening and data extraction

The authors independently screened the literature and extracted data according to the inclusion/exclusion criteria. Discrepancies were resolved through discussion. Electronic databases searched included PubMed, Web of Science, and Google Scholar. Searches were restricted to human studies published between January 2005 and September 2025. RBP4 was only identified in 2005 as an adipokine linked to insulin resistance and metabolic dysfunction, therefore, studied published before this date were not included [7] (Table 1). No language restrictions were initially applied during the search phase, but only studies published in English were selected for final analysis. Bibliographies of all eligible studies were hand-searched to identify additional references. Search outputs were exported into reference management software (EndNote X9) for de-duplication and systematic screening, followed by title/abstract then full-text screening to exclude irrelevant studies. The extracted study characteristics included: (a) Basic characteristics: first author, publication year, country, study design, sample size, participants age and gender distribution; (b) Intervention characteristics: exercise modality, intervention duration, frequency, period, and outcome measures**.**

### Study selection and data collection

**Fig. 1:**
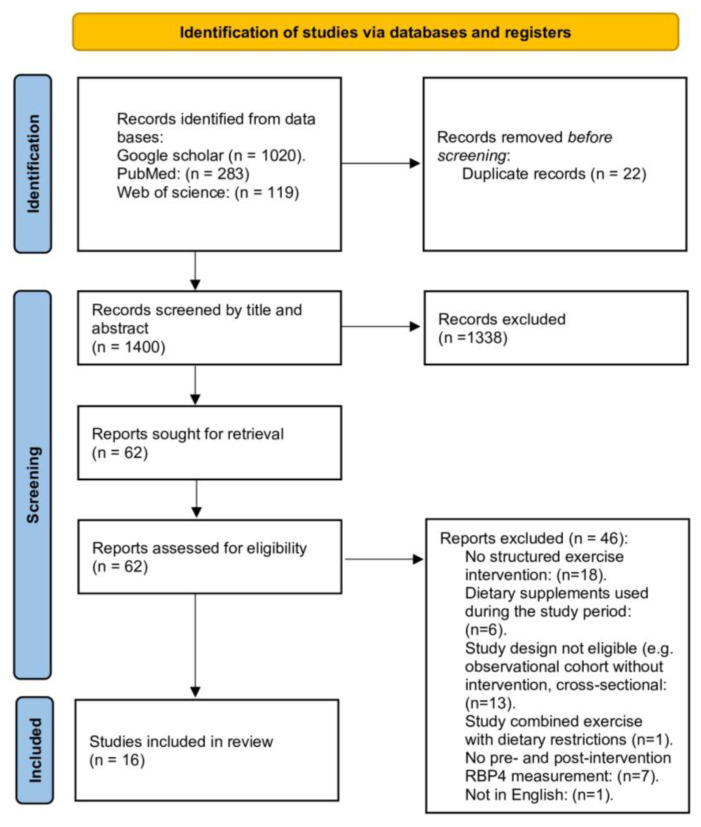
PRISMA flowchart of the review process

The inclusion criteria followed the PICOS framework: (a) Participants: Healthy individuals OR individuals with metabolic disorders (e.g., obesity, insulin resistance); (b) intervention: Physical training (aerobic, resistance, endurance); (c) control group OR pre-training levels; (d) outcomes: changes in circulating RBP4 levels (serum/plasma). Exclusion criteria: we excluded: (a) non-human (animal or in vitro) studies and participants < 18 years; (b) interventions limited to diet, drugs, or supplements without a clear exercise component were not considered, nor were studies combining exercise with dietary manipulation; (c) RBP4 was not measured both pre- and post-intervention, or if only was reported in a figure/graph without providing numeric values; (d) observational, cross-sectional, abstract-only, and review articles; (e) unretrievable full texts articles. The two authors independently screened all retrieved studies, first by title and abstract and then by full text. Based on this process, 16 studies met the eligibility criteria and were included in the final analysis (Fig. 1).

### Quality assessment

The quality of all articles was assessed using the Joanna Briggs Institute (Institute JB approach available at https://joannabriggs.org/critical-appraisal-tools) [32–34]. Quality assessment was carried out according to study design by the two authors, with any disagreements resolved through consensus. Most studies were of moderate to high quality; detailed assessment is available on the Supplementary Table S1.

### Data synthesis

Given the marked heterogeneity in population characteristics, exercise programs, follow-up durations, and statistical reporting, meta-analysis was not performed, and data were synthesized narratively. Studies were grouped and compared by population health status (healthy, obese, diabetic, metabolic syndrome) and by intervention type (aerobic, resistance, combined, and acute training).

## Results

### Intervention characteristics

Of the included studies, six were randomized controlled trials (RCTs) [21,24,25,35–37]. Other studies applied either quasi-experimental or semi-experimental approaches with non-randomized controls [23,26,28,29, 38,39]. The remaining studies employed within-subject longitudinal pre-post designs without controls [22,27,40,41]. Detailed baseline characteristics of these studies are presented in Table 2.

### Baseline circulating RBP4 levels

Baseline circulating RBP4 concentrations varied across study populations. In healthy individuals, mean levels were 41.7 ± 10.6 μg/mL [22,28,35,36,38]. Among patients with T2DM or metabolic syndrome, baseline concentrations were higher, averaging 67.7 ± 10.3 μg/mL [21,24,39]. Obese individuals without metabolic syndrome showed intermediate values of 58.4 ± 10μg/mL [28, 37, 40, 41]. In two studies RBP4 Data were excluded due to implausibly high values [23, 26]. For obese individuals with metabolic syndrome, baseline RBP4 levels were not reported in standardized units and therefore could not be included [25].

### Effects of structured exercise interventions on circulating RBP4 levels

Exercise interventions produced variable effects on circulating RBP4, depending on both the exercise modality and the population studied. Aerobic-based programs including treadmill walking, cycling, brisk walking, aqua training, and combined aerobic-resistance protocols were associated with significant reductions in RBP4 among healthy participants [22, 29], obese individuals [27, 41], women with T2DM [21, 23, 24], and those with metabolic syndrome [25]. Furthermore, resistance training reduced RBP4 levels in T2DM populations [21]. In contrast, other studies reported no significant effects of aerobic exercise on RBP4 levels in healthy cohorts [36, 38] and obese subjects [28, 37]. Moreover, neither acute cardiopulmonary stress testing nor a single session of resistance exercise reduced circulating RBP4 levels [35, 39]. Collectively, these findings suggest that while exercise has the potential to modulate RBP4, its effects are context-dependent and most apparent in metabolically compromised groups.

## Discussion

### Association of RBP4 with insulin sensitivity, glucose homeostasis, and obesity

The role of RBP4 in insulin resistance and glucose homeostasis was first identified by Barbara Kahns group through a series of mouse studies conducted between 1996 and 2005 [7, 42, 43]. Subsequent human studies reporting elevated RBP4 levels in individuals with T2DM [10, 11] led to the proposal that RBP4 influences insulin sensitivity by acting on skeletal muscle and/or the liver [44]. With regard to obesity, RBP4 has been shown to positively correlate with body mass index (BMI) in both healthy and diabetic populations [7, 8]. Moreover, weight reduction interventions have been found to reduce circulating RBP4 levels [18, 45, 46]. Taken together, these findings suggest that training-induced weight loss and improvements in insulin sensitivity may, at least in part, be mediated through reductions in circulating RBP4. However, the evidence summarized in this review indicates that the impact of exercise training on circulating RBP4 remains inconclusive. This does not necessarily imply that exercise fails to reduce RBP4, but rather that the variability may reflect differences in training modalities, baseline RBP4 levels, and population characteristics.

### Baseline differences in circulating RBP4 levels among healthy, obese, and T2DM/metabolic syndrome populations

Baseline RBP4 concentrations differed significantly by population health status. Healthy individuals consistently exhibited the lowest values, whereas obese individuals without metabolic syndrome had moderately elevated levels. The highest concentrations were observed in patients with T2DM or metabolic syndrome, aligning with the known association between RBP4, insulin resistance, and metabolic dysfunction [7, 8]. These findings support the role of RBP4 as a potential biomarker of metabolic dysfunction and highlight its ability to discriminate between metabolically healthy and at-risk populations; however, confirmation is still needed. Further large-scale studies are required to confirm the association between circulating RBP4 levels and metabolic status.

### Effects of different exercise modalities on RBP4 levels

Results of the analyzed studies suggest that exercise-induced reduction of circulating RBP4 levels is context-dependent, with more reduction observed in metabolically compromised groups. This may be explained by the higher baseline RBP4 concentrations or by dysregulated regulatory mechanisms in these groups [7,8]. On the other hand, in obese individuals, the effects of exercise interventions on circulating RBP4 levels were inconsistent. This may be explained by variability in adiposity distribution, dietary control, or exercise intensity. In healthy subjects, reductions were even less consistent. This suggests that RBP4 may be less responsive to exercise in metabolically healthy individuals or that low baseline levels of RBP4 masked any training effects.

### Limitations and future directions

Methodological differences across studies, such as small sample sizes, inconsistent randomization, and variability in RBP4 assay techniques and reporting units, precluded a quantitative meta-analysis. These issues also limit the broader applicability of the findings. Future research should prioritize large randomized controlled trials. Mechanistic studies are also needed to clarify how exercise modulates RBP4. This is essential to establish RBP4 as a reliable biomarker of insulin sensitivity and to clarify the impact of exercise on its circulating levels.

## Conclusion

Exercise interventions appear to reduce circulating RBP4, particularly in metabolically compromised groups. However, findings remain inconsistent across populations and study designs. This is likely driven by variations in study design, exercise modality, intensity, and duration. Overall, these results suggest that exercise has the potential to modulate RBP4, but confirmation requires additional well-designed, large-scale studies.

## Supplementary Information



## Figures and Tables

**Table 1:** Search string

**Pubmed (283)**	(((physical training) OR (exercise)) AND (Retinol Binding Protein 4)) OR (RBP4 circulating levels)		
**Google Scholar (1020)**	(((ALL=(physical training)) OR ALL=(exercise)) AND ALL=(Retinol Binding Protein 4)) OR ALL=(RBP4 circulating levels)		
**Web of Science (119)**	**No.**	**Search Strategy**	**Results**
	1	ALL=((((Physical Training[Mesh]) OR Exercise[Mesh]) OR (exercise)) OR (training))	2,183,204
	2	ALL=((Retinol-Binding Proteins[Mesh]) OR (Retinol Binding Protein 4) OR (RBP4) OR (RBP4 circulating levels))	2,073
	3	1 AND 2	119

**Table 2:** Overview of analyzed studies showing the effects of different exercise modalities on circulating RBP4 level

No	Author/Year	Country	Age (years)/ Gender	Study population (SG) (N)	Control group (CG) (N)	Intervention	Intensity/Frequency	RBP4 Assay/unit	Baseline RBP4 (SD)	Post RBP4 (SD)	RBP4 change
**1**	Ahmadi *et al*. (2013) [38]	Iran	24 (4.8) F	Female athletes (FA) (SG) (n = 10)	FA no exercise (CG) (N = 10)	Aerobic exercise	Moderate 45 min; 3 sessions /week	ELISA μg/ml	(SG): 12.5 (3.4) (CG): 11.6 (4.1)	(SG): 12.9 (3.9) (CG): 112.51.6 (3.3)	(SG): NS. (CG): NS
**2**	Aoki *et al*. (2012) [39]	Japan	65 (8.1) M and F	Diabetic subjects with (G1) or without nephropathy (G2) M (n = 50); F (n = 12)	NA	Single Cardio-pulmonary exercise stress test	NA	ELISA μg/ml	G1): 53.5 (3.6) (G2): 48.2 (4.3)	(G1): 52.2 (2.9) (G2): 54.3 (4.2)	(G1): Significant increase. (G2): NS
**3**	Besse-Patin *et al*. (2014) [40]	France	35.4 (1.5) M	Obese (n = 12)	NA	Endurance training	8 weeks; 5 sessions/ week; 45–60 min/ session	ELISA μg/ml	43.9 (7.2)	41.4 (7.1)	NS
**4**	Bonab *et al*. (2019) [23]	Iran	57.83 (0.42) F	SG: Diabetic with exercise (n = 22)	CG: Diabetic no exercise (n = 22)	Aerobic aqua training	12 weeks; 3 sessions/ week; 60 min/ session	ELISA mm/l	SG: 247.71 (0.55). CG: 248.5 (4.25).	SG: 207.22 (0.73). CG: 245.6 (0.44)	SG: Significant reduction. CG: NS.
**5**	Ghorbanian *et al*. (2023) [25]	Iran	SG: 53.8 (10) M CG: 53.4 (0.9) M	SG: Metabolic syndrome with exercise (n = 10).	CG: Metabolic syndrome without exercise (n = 10).	Aerobic training	12 weeks; 3 sessions/ week; 45 min/ session	ELISA μg/ml	SG: 9.01 (1.10). CG: 9.72 (1.23).	SG: 8.02 (1.09). CG: 9.60 (1.17).	SG: Significant reduction. CG: NS.
**6**	Lim *et al*., (2008) [22]	South Korea	G1: 22.4 (2.8) F. G2: 59.8 (5.9). F	Healthy. G1: (n = 36); G2: (n = 38).	NA	Aerobic training	10 weeks; 3 sessions/ week; 60 min/ session	ELISA μg/ml	G1: 40.7 (12.2). G2: 48.1 (15.3).	G1: 37.7 (9.6). G2: 38 (9.3).	G1: NS. G2: Significant reduction.
**7**	Taghian *et al*. (2014) [26]	Iran	37 (9.89) F.	SG: Obese (n = 10).	CG: Obese with no exercise (n = 10).	Aerobic training	12 weeks; 3 session/ week; 60 min/ session	ELISA pg/ml	SG: 883.3 (457.96). CG: 527.6 (442.9).	SG: 374.5 (469.23). CG: 790.2 (458.6).	SG: Significant reduction. CG: NS.
**8**	Ku *et al*. (2010) [21]	South Korea	G1: 57.8 (8.1) F. G2: 55.7 (6.2) F. G3: 55.7 (7) F.	Obese with T2DM with resistance exercise (G2) (n = 13) or aerobic exercise (G3) (n = 15)	G1: Obese with T2DM with no exercise (n = 16).	G2: Resistance training. G3: Aerobic training.	12 weeks; 5 sessions/ week; 60 min/ session	pg/ml	G1: 95 (20.5); G2: 98.5 (28.8); G3: 87 (25.4).	G1: 96.2 (28.7); G2: 82.1 (27.1); G3: 84.7 (15.3).	G1: NS; G2: significant reduction; G3: NS.
**9**	Choi *et al*. (2013) [27]	South Korea	50.6 (15.1)	Obese (n = 76)	NA	Combined aerobic and resistance training	12 weeks; 5 sessions/ week; 45 min/ session	ELISA μg/ml	94.5 (25.6)	61.8 (20.7)	Significant reduction
**10**	Bonab and Dastah (2023) [24]	Iran	SG: 54 (12) F. CG: 54.5 (0.7) F.	SG: T2DM (n = 20)	Healthy (n = 20).	Aerobic aqua training	12 weeks; 3 sessions/ week; 60 min/ session	ELISA μg/ml	SG: 90.36 (15.23). CG: 87.21 (16.31).	SG: 70.98 (11.42). CG: 88.47 (13.44)	SG: Significant reduction. CG: NS.
**11**	Numao *et al*. (2012) [41]	Japan	48 (2) M.	Obese (n = 29)	NA	Aerobic training	12 weeks; 3 sessions/ week; 45–60	ELISA μg/ml	55.4 (3.4)	49.9 (2.8)	Significant reduction.
**12**	Choi *et al*. (2009) [28]	South Korea	46.7 (7.5) F.	Obese (n = 30)	NA	Aerobic training	12 weeks; 5 sessions/ week; 45 min	ELISA mg/L	33.5 (13.4).	34.8 (11)	NS
**13**	Doğru *et al*. (2016) [29]	Turkey	MTWG: 38 F; BWG: 44 F; CG: 38.5 F	Healthy (MTWG: n = 11; BWG: n = 12)	Healthy with no exercise (n = 12)	Moderate (MTWG) or brisk tempo waling (BWG)	8 weeks; 3 session/ week; 30–50 min/ session	ELISA ng/ml	MTWG: 61.4; BWG: 65.4; CG: 55.4	MTWG: 49.5; BWG: 51.2; CG: 59	MTWG and BWG: Significant reduction. CG: NS
**14**	Mansouri *et al*. (2011) [35]	Iran	SG1: 22.4 (1.8), CG1: 22 (0.9); SG2: 21.7 (0.9), CG2: 24.6 (1.6). All M.	SG1: healthy athlete (n = 10). SG2: healthy sedentary (n = 8).	CG1: healthy athlete (n= 9) with no exercise. CG2: Healthy sedentary (n = 7) with no exercise.	Resistance training	One session	ELISA μg/ml	SG1: 46.5 (18); CG1: 52 (23); G2S: 54 (10); CG2: 46 (19).	SG1: 44 (1.4); CG1: 41 (27); SG2: 46.5 (17); CG2: 42.8 (19).	NS in all groups.
**15**	Ratajczak *et al*. (2024) [37]	Poland	SG: 33.4 (4.5) F; CG: 32 (5) F.	Women with IR (n = 19)	(n = 8)	Combined strength and endurance training	12 weeks; 3 session/ week; 33 min/ session	RBP4 test by IDN mg/ mL	SG: 62 (24); CG: 78.6 (26)	SG: 55.85 (25); CG: 71.7 (9.6)	NS
**16**	Moghadasi *et al*. (2013) [36]	Iran	24 (4.3) F.	Healthy karate players (n = 10)	(n = 10)	High intensity aerobic training	8 weeks; 3 sessions/ week; 45 min/ session	ELISA μg/ml	SG: 11.1 (3.2); CG: 11.6 (4.1)	SG: 12 (2); CG: 12.5 (3.3)	NS

CG control group, SG study group, ELISA enzyme linked immunosorbent assay kit, NS not significant, IR insulin resistance, IDN Immun Diagnostik

## Abbreviations

T2DM: Type-1 diabetes mellitus
RBP4: Retinol-binding protein 4
PRISMA: Preferred reporting items for systematic reviews and meta-analyses
ELISA: Enzyme-linked immunosorbent assay
BMI: Body mass index
