# Supplementary material for: The Impact of Physical Training on Circulating Retinol-Binding Protein 4: A Systematic Review
Source: Physiol Res. 2026 Apr 1;75(2):193–201. doi: 10.33549/physiolres.935730 (PMC13225445; doi:10.33549/physiolres.935730)
Supplement: Supplementary file 1 [file PR75_193_Supplementary_Table_S1.pdf]

Physiol. Res. 75: 193-201, 2026 *https://doi.org/10.33549/physiolres.935730*

REVIEW

The Impact of Physical Training on Circulating Retinol-Binding Protein 4: A Systematic Review

Zaki ALSAHAFI[1,2], Ahmaed BAASHAR[1,2]

Supplementary Table S1: JBI assessment of the analyzed studies

	**JBI Randomized Controlled Trials (Q1-Q13)**												
**Authors (date)**	Q1	Q2	Q3	Q4	Q5	Q6	Q7	Q8	Q9	Q10	Q11	Q12	Q13
**Ghorbanian *et al.*, (2023) [25]**	Y	U	Y	Y	Y	U	Y	Y	Y	Y	Y	Y	Y
**Ku *et al.*, (2010) [21]**	Y	U	N	Y	Y	U	Y	NA	Y	Y	U	Y	Y
**Bonab and Dastah 2023 [24]**	Y	U	Y	U	U	U	Y	Y	Y	Y	U	Y	Y
**Mansouri *et al.*, (2011) [35]**	Y	U	Y	N	U	Y	U	Y	Y	Y	Y	Y	Y
**Ratajczak *et al.*, (2024) [37]**	Y	U	Y	U	U	Y	U	Y	Y	Y	Y	Y	Y
**Moghadasi *et al.*, (2013) [36]**	Y	U	Y	U	U	Y	U	Y	Y	Y	Y	Y	Y

**JBI Quasi-Experimental (Non-randomized) (Q1-Q9)**									
	Q1	Q2	Q3	Q4	Q5	Q6	Q7	Q8	Q9
**Ahmadi *et al.*, (2013) [38]**	Y	Y	Y	Y	Y	Y	U	U	Y
**Aoki *et al.*, (2012) [39]**	Y	Y	Y	Y	Y	Y	U	U	Y
**Bonab *et al.*, (2019) [23]**	Y	Y	Y	Y	Y	Y	U	U	Y
**Choi *et al.*, (2009) [28]**	Y	Y	Y	Y	Y	Y	Y	Y	Y
**Doğru *et al.*, (2016) [29]**	Y	Y	Y	Y	Y	U	U	U	Y
**Taghian *et al.*, (2014) [26]**	Y	Y	Y	Y	Y	U	U	U	Y

**JBI Analytical Quasi-Experimental (Pre–Post) (Q1-Q8)**								
	Q1	Q2	Q3	Q4	Q5	Q6	Q7	Q8
**Besse-Patin *et al.*, (2014) [40]**	Y	Y	Y	Y	Y	Y	Y	Y
**Choi *et al.*, (2013) [27]**	Y	Y	U	Y	U	U	Y	Y
**Numao *et al.*, (2012) [41]**	Y	Y	U	Y	U	Y	Y	Y
**Lim *et al.*, (2008) [22]**	Y	Y	Y	N	Y	Y	U	Y
